# Long-term aspirin administration suppresses inflammation in diabetic cystopathy

**DOI:** 10.18632/aging.205021

**Published:** 2023-09-12

**Authors:** Huifang Du, Feihong Xu, Jingxuan Liu, Jiakui Zhang, Yinhua Qin, Youqian Xu, Ning Li

**Affiliations:** 1 Department of Urology, Fourth Affiliated Hospital, China Medical University, Shenyang, China; 2 Department of Anatomy, Army Medical University (Third Military Medical University), Chongqing, China

**Keywords:** diabetic cystopathy, aspirin, inflammatory, bladder, detrusor smooth muscle

## Abstract

Diabetic cystopathy (DCP) is one of the most common and troublesome urologic complications of diabetes mellitus, characterized by chronic low-grade inflammatory response. However, the correlation between inflammation and disease progression remains ambiguous and effective drugs interventions remain deficient. Herein, during 12-week study, 48 male Sprague-Dawley rats were randomly assigned to four groups: negative control (NC), NC treated with aspirin (NC+Aspirin), DCP, and DCP treated with aspirin (DCP+Aspirin). Type 1 diabetes mellitus was established by intraperitoneal injection of streptozotocin (65 mg/kg). After 2 weeks modeling, the rats in treatment groups received daily oral aspirin (100 mg/kg/d). After 10 weeks of treatment, aspirin ameliorated pathological weight loss and bladder weight increase in diabetic rats, accompanied by a 16.5% decrease in blood glucose concentrations. H&E, Masson, immunohistochemistry and transmission electron microscopy revealed that a dilated bladder with thickened detrusor smooth muscle (DSM) layer, inflammatory infiltration, fibrosis and ultrastructural damage were observed in diabetic rats, which were obviously ameliorated by aspirin. The dynamic investigations at 4, 7 and 10 weeks revealed inflammation gradually increased as the disease progresses. After 10 weeks of treatment, the expression of TNF-α, IL-1β, IL-6, and NF-κB has been decreased to 78%, 39.7%, 44.1%, 33.3% at mRNA level and 67.6%, 76.7%, 71.4%, 67.1% at protein level, respectively (DCP+Aspirin vs. DCP, p < 0.01). Aspirin partially restored the increased expression of inflammatory mediators in bladder DSM of diabetic rats. The study provided insight into long-term medication therapies, indicating that aspirin might serve as a potential strategy for DCP treatment.

## INTRODUCTION

Diabetic cystopathy (DCP) is one of the most common and troublesome urologic complications of diabetes mellitus, has a profound impact on the quality of life [1]. DCP affects up to 80% of all patients with diabetes mellitus, characterized by symptoms of overactive bladder and underactive bladder, which causes a range of storage and voiding problems clinically [2–4]. DCP has been traditionally described as a triad of decreased bladder sensation, increased bladder compliance and capacity, and impaired detrusor contractility. The pathophysiology mechanism of DCP is multifactorial, such as the disorder of detrusor smooth muscle (DSM), urothelium, nerves, bladder mucosa and urethra [5]. Xu et al*.* found that inflammation plays important but distinctive roles in the induction of DCP [6]. A more complete understanding of the natural history of DCP and temporal inflammation variations in its pathophysiology is increasingly important.

DCP includes time-dependent and mixed manifestations. The varied clinical presentation may be related to the duration of diabetes. Previous research has revealed a transition from a compensated to a decompensated state in STZ-induced diabetic rats begins 9 to 12 weeks after induction [7–10]. Nevertheless, there has not as yet, been a large-scale trial to examine the definite role of inflammation on the risk of developing diabetes. During the early compensatory hypertrophy bladder caused by overactive bladder, if active efforts are made, the function of bladder can be reversed. Therefore, it is an important research problem to strengthen the research into the early bladder damage at present. Generally, the treatment for DCP is basically conservative, can be categorized into behavioral, pharmacological or surgical. Among them, antimuscarinics are the chief clinically well-established approach for improving symptoms of overactive bladder in the early stage of bladder compensation. Nonetheless, they may be detrimental in the late decompensated state. Additionally, the existing medical therapies have varying degrees of drug resistance and adverse reactions, which limit their wide applications [11, 12]. Recent clinical and experimental evidence supports the inflammatory hypothesis [13–15] that in the process of chronic low-grade inflammation, the release of cytokines and pathologic mediators seems to be involved in the development of cardiovascular, renal, and ophthalmological complications of diabetes mellitus [16–19]. Accordingly, early aggressive treatment or intervention plays a key role in improving DCP and our research endeavors to identify a remedy to investigate a therapeutic strategy to improve bladder by targeting inflammation.

Aspirin is a commonly used non-steroidal anti-inflammatory drug (NSAID) in clinical practice. Aspirin as a cyclooxygenase and NF-κB inhibitor, plays an anti-inflammatory role by inhibiting the synthesis of prostaglandins, inhibiting the aggregation of leukocytes and reducing the formation of bradykinin [20, 21]. High-dose aspirin has exhibited impressive results in reducing fasting plasma glucose concentration and improving insulin resistance by inhibiting NF-κB activation and TNF-α level in STZ-induced type 2 diabetic rats [22, 23]. Aspirin can attenuate vascular smooth muscle cell migration via the cyclic adenosine monophosphate/protein kinase A (cAMP/PKA) pathway [24]. Daily administration of low-dose aspirin has proved to be beneficial in preventing recurrent cardiovascular events in patients who had diabetes [25–27]. Additionally, previous studies have reported that high-dose aspirin exhibited impressive potential in clinics for protecting against diabetic cataract [28], diabetic encephalopathy [29], diabetic nephropathy and other related disorders or complications of diabetes, without inducing serious undesirable symptoms [20]. However, there is no clinical data to reveal the efficacy of aspirin on improving bladder inflammation of DCP. Herein, this study aimed to explore the inflammation and morphological alterations in different development processes of DCP and the potential of aspirin in attenuating inflammatory reactions in bladder tissue by establishing an STZ-induced type 1 diabetic rat model.

## RESULTS

### General characteristics

**Figure 1:**
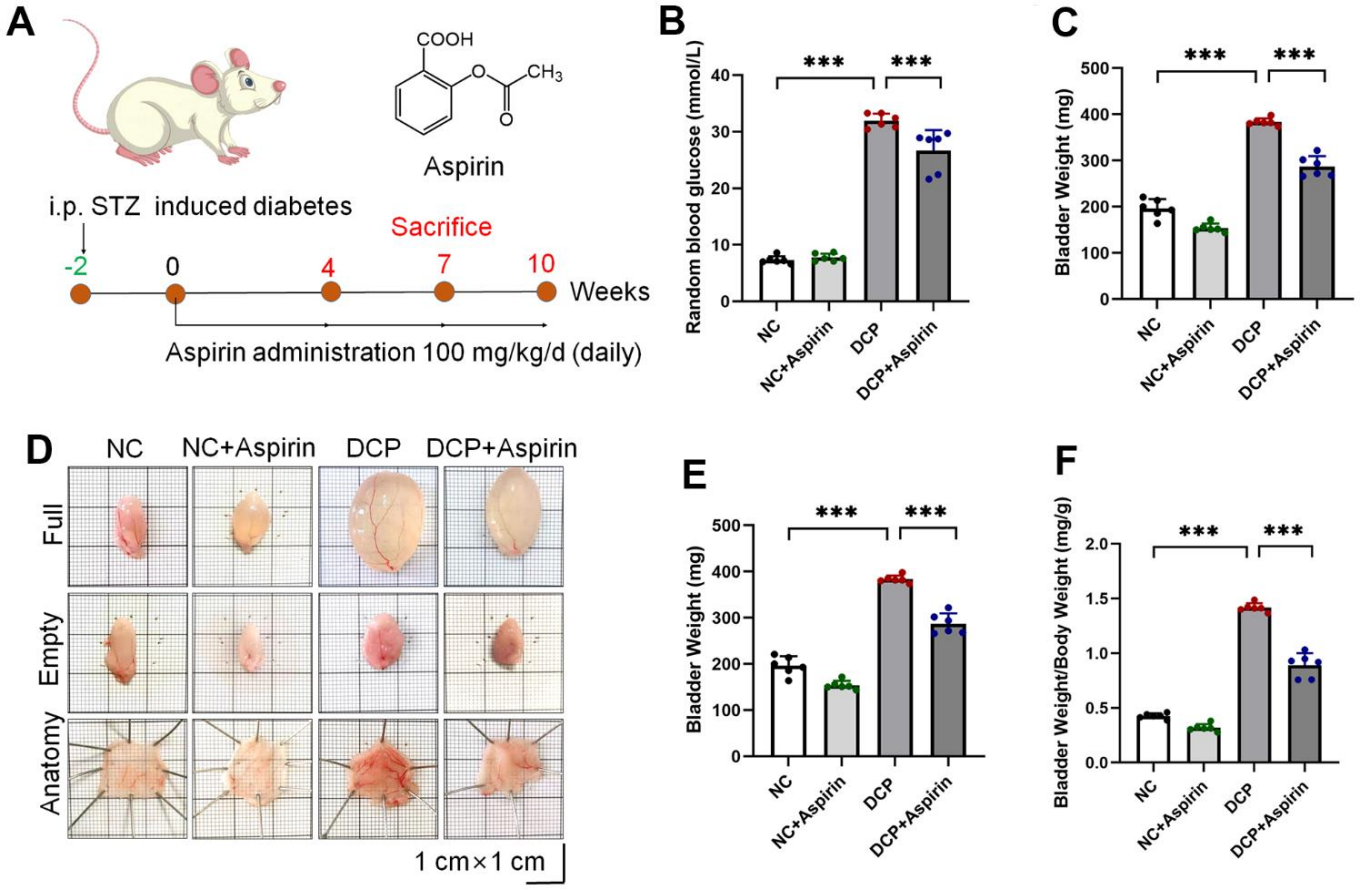
**STZ-induced diabetic rats intragastric administration with aspirin.** (**A**) Schematic illustration of the experimental timeline and the chemical structure of aspirin. (**B**) Random blood glucose (**C**) Body weight. (**D**) Representative macroscopic findings of bladder specimens. (**E**) Bladder weight in the empty state. (**F**) Relative bladder weight (bladder weight/body weight). Data were presented as mean ± SD. N=6. (ns, no significant; *, P < 0.05; **, P < 0.01; ***, P < 0.001; NC vs DCP; DCP vs DCP+Aspirin).

The rat model of type 1 diabetes was successfully established according to the design project (Figure 1A, and Supplementary Figure 1A). The random blood glucose of diabetic rats was 3-4 times higher than that of normal rats (Figure 1B). After 10 weeks of treatment, the elevation of random blood glucose caused by diabetes was lowered by aspirin. Aspirin had hypoglycemic activity, causing an approximately 16.5% decrease in blood glucose concentrations (DCP+Aspirin vs. DCP, p < 0.001). The body weight of normal rats gradually increased, while diabetes rats developed typical symptoms such as polydipsia, polyphagia, polyuria and weight loss (Supplementary Figure 1B). After 10 weeks of treatment, aspirin improved the weight loss of diabetes rats (Figure 1C). Gross assessment preliminarily showed that the bladder wall was thicker in the DCP group, and aspirin treatment reduced bladder thickening (Figure 1D). The absolute and relative bladder weight of the DCP group were increased over those of controls by approximately 93% (Figure 1E) and 265% (Figure 1F) respectively. Aspirin treatment partially reversed this change to approximately 75% and 63% (DCP+Aspirin vs. DCP, p < 0.001).

### Histological changes in STZ-induced diabetic rats after 10 weeks of aspirin administration

**Figure 2:**
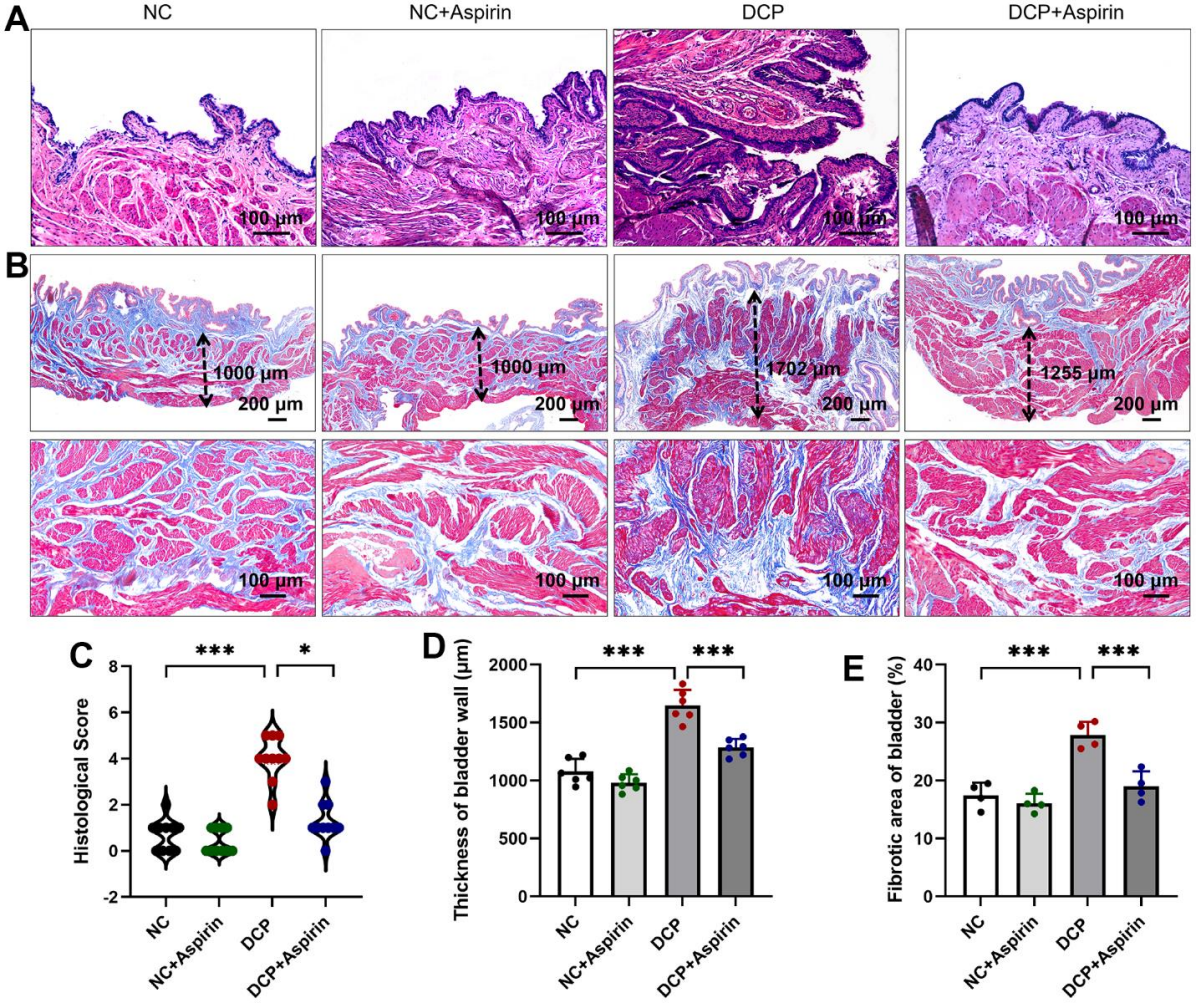
**The effect of aspirin on bladder injury in STZ-induced diabetic rats after 10 weeks administration.** (**A**) H&E-stained micrographs of histological features of rat bladder sections. (**B**) Masson-stained micrographs. The dotted arrowheads indicate the thickness of the bladder wall. (**C**) Histological score based on inflammation. (**D**) Statistical analysis of the thickness of bladder wall. (**E**) Morphological evaluation was performed with measurement of bladder fibrosis and collagen content. Data were presented as mean ± SD. N=6. (***, P < 0.001; NC vs DCP; DCP vs DCP+Aspirin).

As shown in Figure 2A, H&E results showed inflammatory cell infiltration, edema, hemorrhage and capillary proliferation of mucosa and lamina propria occurred in the bladder tissue of the DCP group, consistent with previous research. Bladder wall thickness and collagen volume fraction in the DSM tissue were measured on Masson-stained images (Figure 2B). Inflammation and injury were prominently ameliorated after 10 weeks of aspirin treatment (Figure 2C). Morphometric analysis revealed bladder wall thickness (Figure 2D) and collagen volume fraction (Figure 2E) in the DCP group were up to 1.5 times compared with the NC group. After 10 weeks of aspirin treatment, the thickness of the bladder wall was reduced by 78% (DCP+Aspirin vs. DCP, p < 0.001), and the ratio of smooth muscle/collagen in the detrusor layer of the bladder was also reduced by 68% (DCP+Aspirin vs. DCP, p < 0.001). Aspirin attenuated bladder hypertrophy in STZ-induced diabetic rats.

### The effect of aspirin on ultrastructure of the DSM in STZ-induced diabetic rat

**Figure 3:**
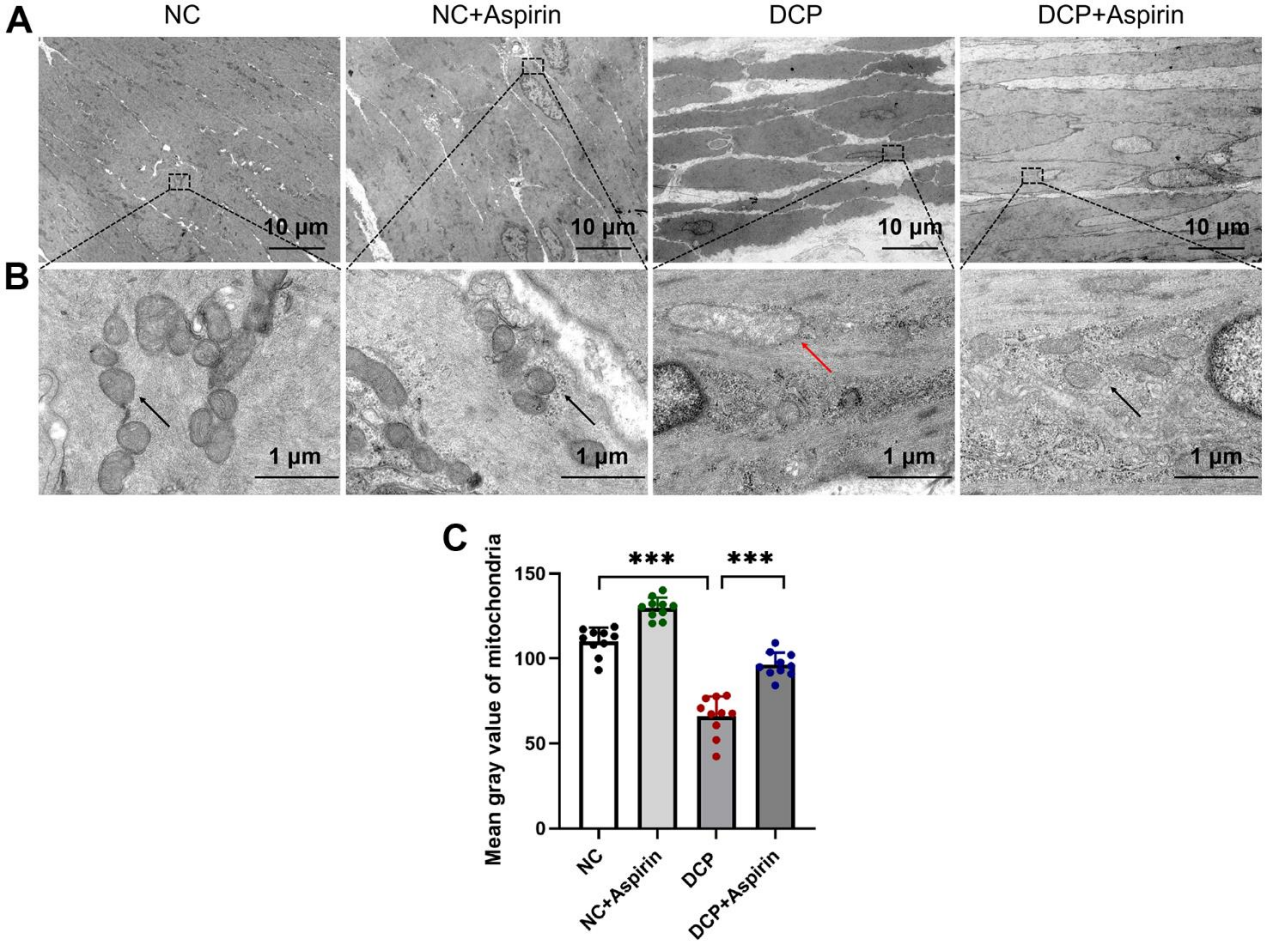
**Transmission electron microscopy (TEM) of bladder DSM tissue after 10 weeks of aspirin administration.** Organelles such as nucleus, mitochondria, and rough endoplasmic reticulum are seen. (**A**) A representative image from the bladder DSM at a magnification of ×1,000. (**B**) A representative image from the bladder DSM at a magnification of ×15,000. The red arrowheads indicate the swollen mitochondria. (**C**) Statistical analysis of the mitochondrial matrix optical density. Data were presented as mean ± SD. N=6. (***, P < 0.001; NC vs DCP; DCP vs DCP+Aspirin).

Morphometric analysis under the light microscope revealed a remarkable increase in the areas of the detrusor muscle and urothelium in diabetic rats (Figure 2). To take a closer look, the bladder detrusor smooth muscle cells (DSMCs) and fibroblasts were observed under transmission electron microscopy (TEM). The detrusor histology of the DCP group rats was characterized by the presence of thickened and hypertrophied microfibers, some of which were dissolved and necrotic (Figure 3A). Besides that, as shown in Figure 3B, there was severe mitochondrial damage in the DSMCs of the diabetic rat bladder, manifested as increased mitochondrial volume, decreased number of “ridges”, ruptured or even disappeared, and decreased mitochondrial matrix optical density (Figure 3C). In addition, mitochondrial vacuolar degeneration and endoplasmic reticulum swollen was observed. Aspirin repaired the damaged DSMCs by reducing mitochondrial damage.

### The effect of aspirin on histological and inflammation-related factor changes in STZ-induced diabetic rat

**Figure 4:**
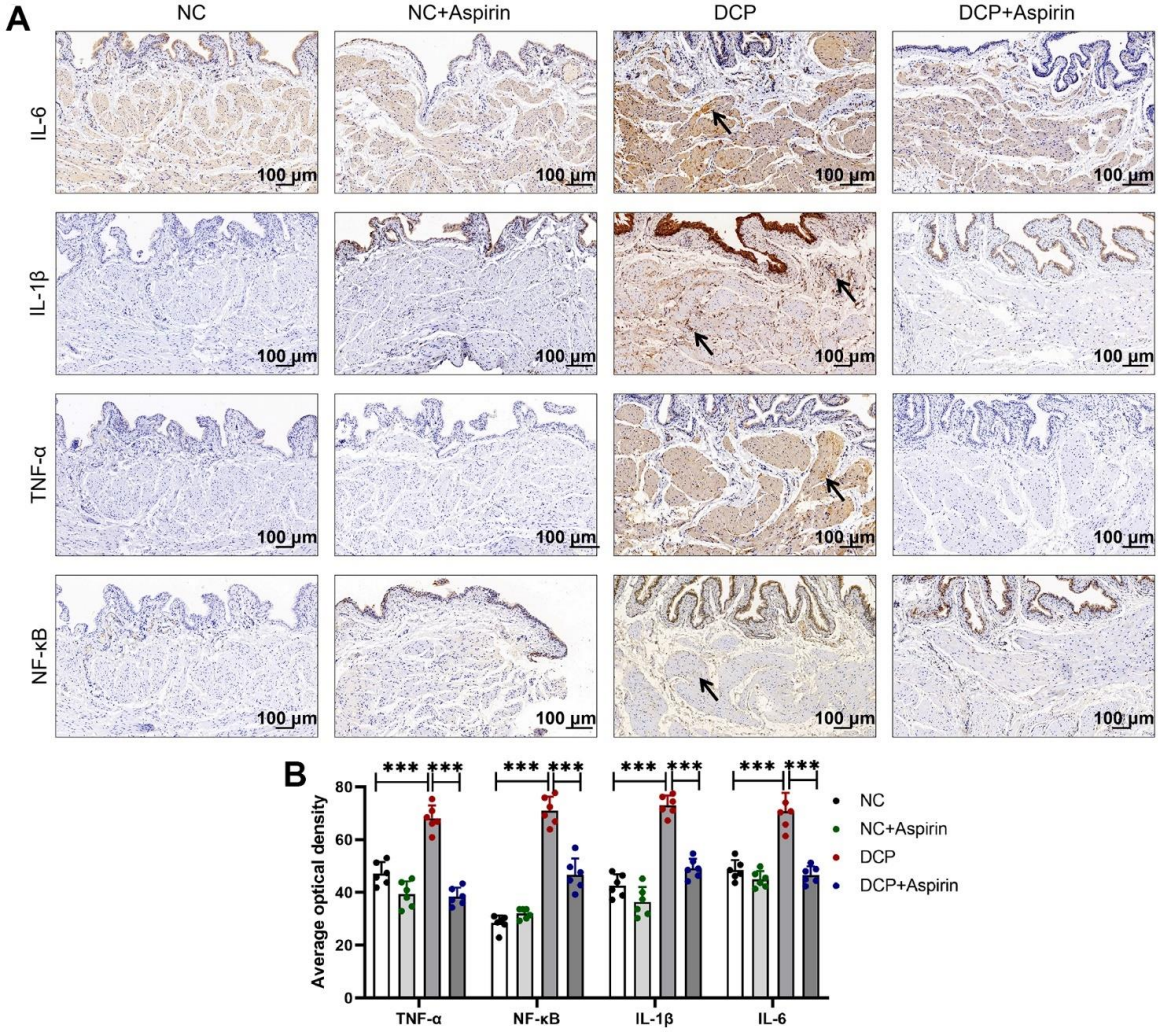
**Immunohistochemistry shows aspirin suppress DSM tissue inflammation in diabetic rats after 10 weeks administration.** (**A**) Immunohistochemistry indicates the expression of inflammatory mediators IL-6, IL-1β, TNF-α, and NF-κB was mainly distributed in the mucosa, lamina propria and meanwhile detrusor muscle. Arrows indicate positive staining (brownish-yellow). (**B**) The average optical density of IL-6, IL-1β, TNF-α, and NF-κB were analyzed using Image J Analysis System. Data were presented as mean±SD. N=6. (***, P < 0.001; NC vs DCP; DCP vs DCP+Aspirin).

Polyuria and impaired insulin signaling induced by disorders of glucose metabolism can increase macrophage infiltration and secrete inflammatory cytokines, leading to local and systemic inflammation [14, 30]. NF-κB is an upstream mediator of cytokine transcription, which plays a key role in regulating the multiple genes involved in the inflammatory response in bladder tissue, such as TNF-α, IL-6, and IL-1β. Immunohistochemical results indicated inflammatory indicators (NC vs. DCP, p < 0.001) NF-κB, TNF-α, IL-6, and IL-1β were increased by 44.3%, 150.1%, 71.5% and 46.0% respectively in the DSM of diabetic rats (Figure 4). Interestingly, the expression of NF-κB, TNF-α, IL-6, and IL-1β in the DCP+aspirin group generally reverted toward normal levels, with a noticeable decrease (DCP vs. DCP+Aspirin, p < 0.001) to 56.5%, 65.9%, 67.2% and 66.0% of the DCP group.

### Hyperglycemia upregulated the inflammatory factor protein activity and mRNA level in the DSM tissue of STZ-induced diabetic rats

**Figure 5:**
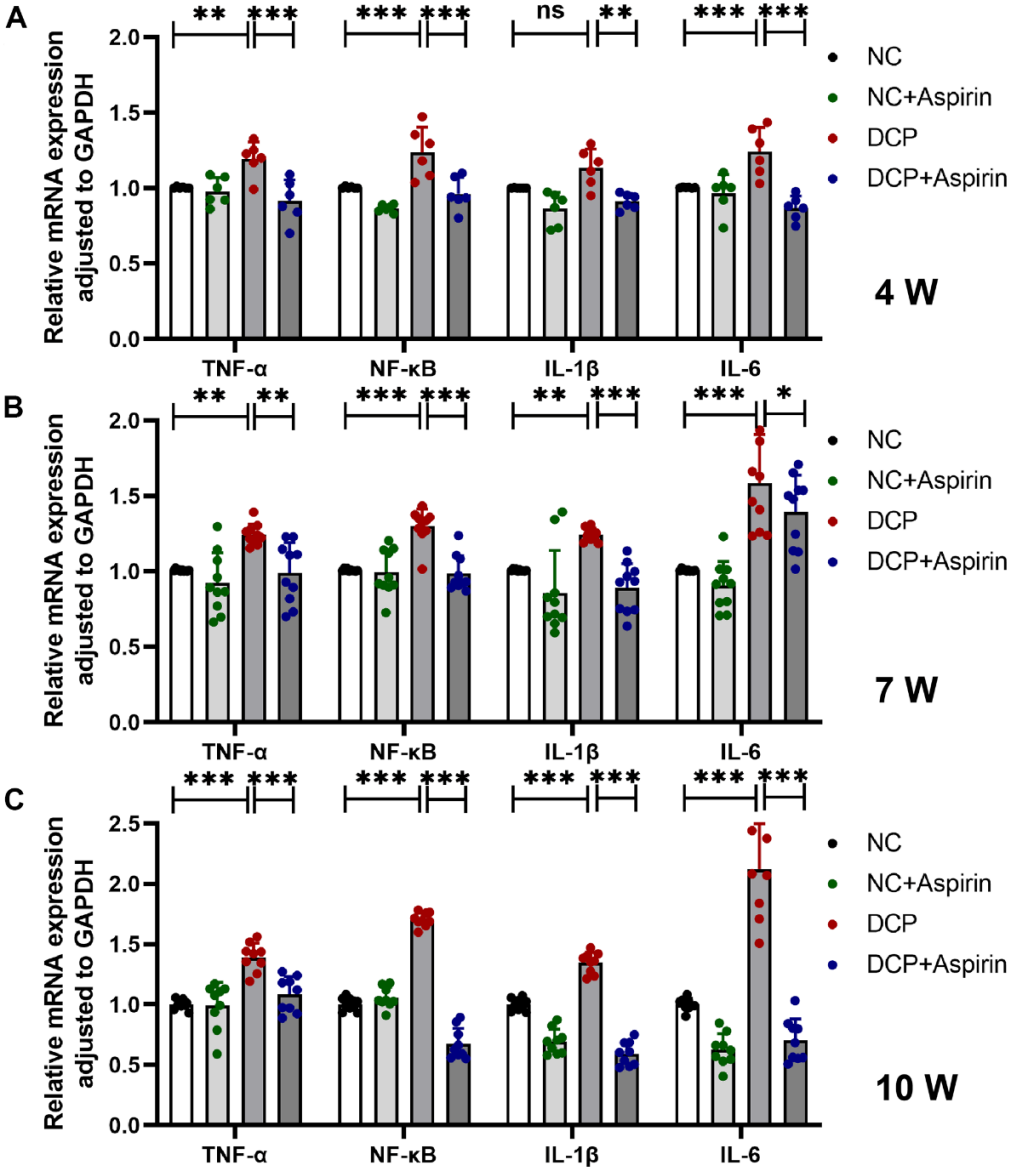
**The mRNA expression levels of TNF-α, NF-κB, IL-1β and IL-6 in bladder DSM tissue of three treatment durations.** (**A**) Statistical analysis after 4 weeks of aspirin treatment. N=3. (**B**) Statistical analysis after 7 weeks of aspirin treatment. N=3. (**C**) Statistical analysis after 10 weeks of aspirin treatment. Data were presented as mean ± SD. N=6. (ns, no significant; *, P < 0.05; **, P < 0.01; ***, P < 0.001; NC vs DCP; DCP vs DCP+Aspirin).

To explore how aspirin treatment prevented bladder injury, we evaluated mRNA and protein expression levels of pro-inflammatory cytokines at different stages of diabetes. As shown in Figure 5A, after 4 weeks of aspirin treatment, the mRNA expression levels of TNF-α, NF-κB, IL-1β, and IL-6 in the DCP group increased by 19.1% (p < 0.01), 23.3% (p < 0.001), 13.2% (p = 0.1087) and 23.6% (p < 0.001) respectively compared with the control group. After 7 weeks, TNF-α, NF-κB, IL-1β and IL-6 in the DCP group were 23.6% (p < 0.01), 29.1% (p < 0.001), 23.5% (p < 0.01) and 57.5% (p < 0.001) higher than those of the control group (Figure 5B). Statistics on 10 weeks increased (p < 0.001) by 39.1%, 70.2%, 34.5% and 111.8%, respectively (Figure 5C). To go along with this, compared with the DCP group, the mRNA expression levels of TNF-α, NF-κB, IL-1β, and IL-6 in the DCP+Aspirin group decreased to 76.7%, 75.3%, 81.9% and 71.6% after 4 weeks of aspirin treatment; 79.5%, 75.7%, 71.6% and 87.9% after 7 weeks of aspirin treatment; 78.0%, 39.7%, 44.1% and 33.3% after 10 weeks of aspirin treatment.

**Figure 6:**
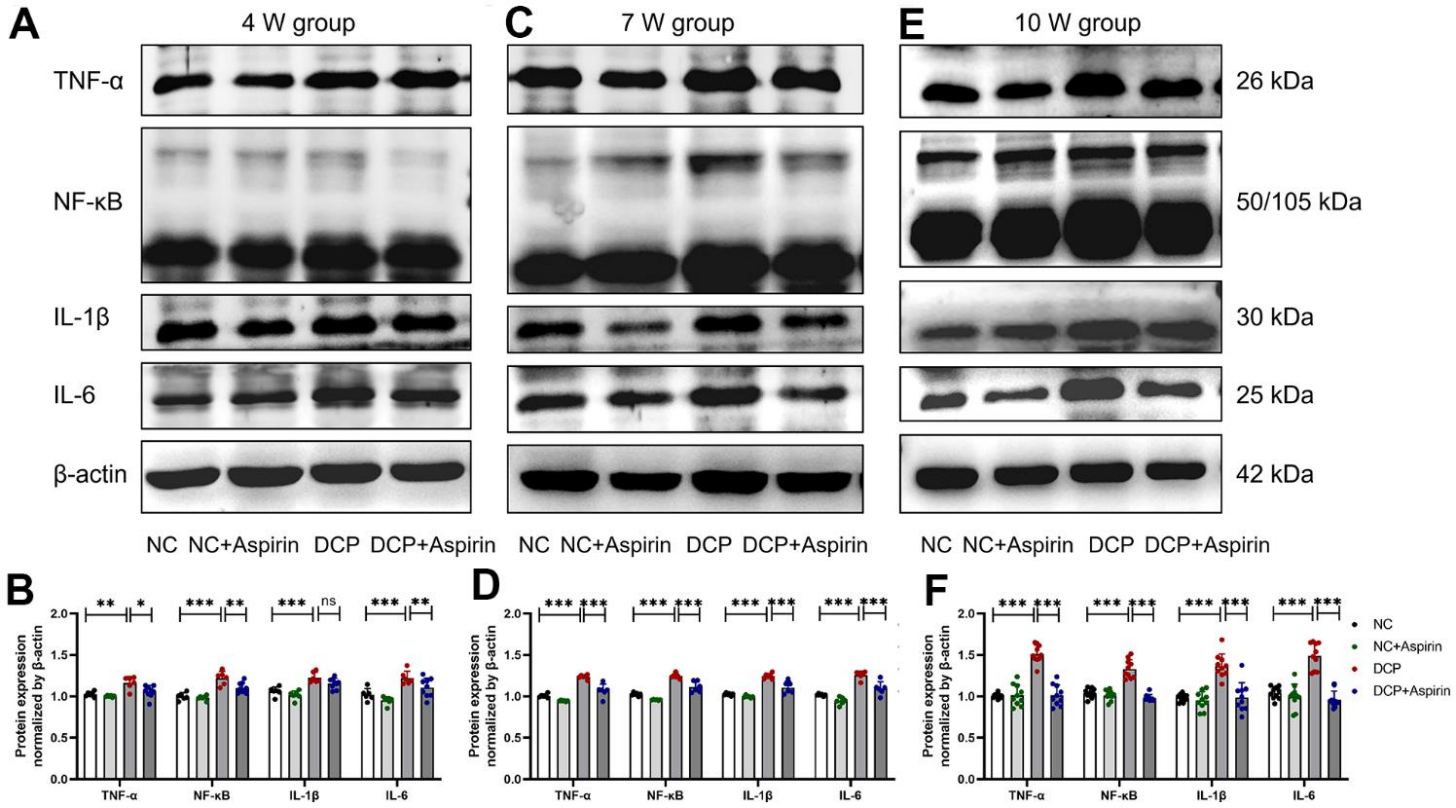
**The protein expression levels of TNF-α, NF-κB, IL-1β and IL-6 in bladder DSM tissue of three treatment durations.** (**A**, **B**) Western blot bands and statistical analysis after 4 weeks of aspirin treatment. N=3. (**C**, **D**) Western blot bands and statistical analysis after 7 weeks of aspirin treatment. N=3. (**E**, **F**) Western blot bands and statistical analysis after 10 weeks of aspirin treatment. Data were presented as mean ± SD. N=6. (ns, no significant; *, P < 0.05; **, P < 0.01; ***, P < 0.001; NC vs DCP; DCP vs DCP+Aspirin).

Then we evaluated the expression of the inflammatory mediators aforementioned at the protein level by western blot assay. As shown in Figure 6, the results evidenced that the protein expression level of TNF-α, NF-κB, IL-1β, and IL-6 in the DSM tissue of the DCP group (p < 0.01) was remarkedly higher than that of the control group. After 4 weeks of aspirin treatment, the up-regulated rate (NC vs. DCP, p < 0.01) was 14.0%, 21.1%, 16.7% and 17.9%. After 7 weeks, the up-regulated rate (NC vs. DCP, p < 0.001) reached 22.7%, 21.1%, 21.0% and 22.9%. After 10 weeks, the up-regulated rate (NC vs. DCP, p < 0.001) was up to 50.0%, 28.2%, 38.5% and 43.3%, respectively. Correspondingly, the promotion effect of STZ on the protein expression level of TNF-α, NF-κB, IL-1β, and IL-6 in the DSM tissue of the DCP group was partially restored by aspirin administration. Aspirin postconditioning reduced inflammation to 91.0% (p < 0.05), 90.2% (p < 0.01), 94.0% (p = 0.1563) and 90.1% (p < 0.01) after 4 weeks of treatment; 7 weeks (p < 0.001) to 87.2%, 92.0%, 91.7% and 89.2%; 10 weeks (p < 0.001) to 67.6%, 76.7%, 71.4% and 67.1%. To summarize, the levels of inflammatory cytokines increased gradually with the course of the disease. Aspirin exerts a therapeutic mitigative effect on inflammation formation, which is consistent with the mRNA expression level. The pharmacological inhibition of aspirin may be one of the main mechanisms for inhibiting the release of inflammatory mediators in DSM of diabetic rats.

## DISCUSSION

Diabetes mellitus is a grave and progressive condition characterized by debilitating complications. The prevalence and incidence rate of diabetes mellitus and its complications is increasing rapidly worldwide [31, 32]. Although up to 80% of diabetic patients will suffer from voiding difficulties and urinary symptoms, defined as DCP, the mechanisms of its development are not clear. The low efficacy of current therapeutics and lifestyle interventions combined with high national healthcare costs highlight the need for more research into DCP pathophysiology and novel treatment options. Therefore, we intend to seek a better therapeutic drug by investigating the time course of DCP and concurrent changes in inflammation using an STZ-induced diabetic rat model.

Targeting inflammation currently has potential in diabetes and diabetic complications [16–19]. NSAIDs have effects on urodynamic parameters of normal and cystitis rats [20]. The application researches so far include aspirin, resveratrol, indomethacin, ketoprofen, curcumin and so on [6, 20]. Aspirin, in contrast to ketoprofen or indomethacin, showed a great characterization with less gastrointestinal lesions and might be the NSAIDs treatment of choice for overactive bladder [20]. Meanwhile, different from oral resveratrol [6], in this work, aspirin showed additional advantages in ameliorating pathological weight loss and reducing random blood glucose levels in diabetes rats. The hypoglycemic effect is the differentiated advantage of aspirin over other anti-inflammatory drugs. Better yet, the swollen and degenerated mitochondria observed in the bladder DSM of diabetic rats were partially restored by aspirin, which may be a key point in the amelioration of bladder injury. Accumulating evidence supports aspirin can significantly reduce the risk of digestive tract cancer [33] and plays a pivotal role in the prevention and treatment of cardiovascular disease [25–27] in diabetes. To observe the apparently effect in this work, we took a high-dose for daily administration. However, the benefits of aspirin are often accompanied by adverse effects, especially a significant increase in the risk of bleeding. Although all rats in our experiment remained alive without death, macroscopic flatulence or gastrointestinal bleeding, precision dosing trials and individualized drug regimens should be designed to balance risk and benefit in the further research.

Diabetes can cause pathological alterations in the morphology and structure of bladder tissue. Bladder hypertrophy is a noticeable characteristic in diabetic rats, which is generally consistent with related research results [34, 35]. Quantification of the bladder wall components of diabetic rats revealed the primary source of the increased bladder weight was detrusor muscle and collagen content, with contributions from lamina propria, urothelium and adventitia, which is consistent with previous studies [1, 36]. Increased bladder weight and wall thickness were associated with edema and hemorrhage. An earlier study has shown that diabetes-induced polyuria can stimulate DNA synthesis, which in turn results in increased protein synthesis, causing increased cell mass and hyperplasia [9, 37]. The change in morphology might be a compensatory response to the increased urine output in diabetic rats. Research showed mitochondria in bladder DSMCs of diabetic rats were damaged to varying degrees. These ultrastructural changes can make DSM lose its normal contractile function and reduce the coupling effect, leading to detrusor contractile dysfunction [38].

In addition to bladder hypertrophy and altered tissue composition, H&E staining and immunohistochemistry preliminarily understand the morphologic impairments, such as inflammatory infiltration, edema, bleeding, capillary hyperplasia in the bladder was mainly distributed in the mucosa, lamina propria and DSM tissue. Daneshgari et al. evaluated time-dependent changes in bladder function in STZ-induced diabetic rats based on urodynamic data [7–10]. There has been little systematic effort to reveal the sequential changes of bladder inflammation, despite a profound and lasting impact on DCP. Specifically, we identified key inflammatory mediators and evaluated the effects of aspirin pharmacological treatments. The PCR and western blot results indicated that inflammation alterations are time-dependent and may play an important role in pathological changes. Aspirin decreased STZ-induced referred bladder inflammation after 4, 7 and 10 weeks post-administration and reversed the increased wall thickness, edema, hemorrhage, and macroscopic damage. To summarize, this study reveals temporal inflammation effects and pathological changes of bladder tissue and confirms that aspirin has a therapeutic effect in the STZ-induced type 1 diabetes model.

Taken together, these findings strengthen the case for consideration of long-term aspirin use in DCP treatment. There are still some limitations in this study. To better understand the relevance of bladder hypertrophy in many models of experimental diabetes, it is still necessary to further investigate separately in type 1 and type 2 diabetes [34]. The damage is a gradually changing process, thus the experiment still needs to dynamically observe at longer and more time points. There is a lack of consensus about the balance of risks and benefits associated with long-term aspirin use, particularly on high doses. Considering medication safety, it is essential to guide precision treatments that maximize benefits and minimize risks. The optimal dose for DCP treatment and the precise mechanism of underlying aspirin’s anti-inflammatory effect require further investigation. Intravesical drug delivery can be considered in future studies to decrease harmful side-effects and drug toxicity, and improve drug bioavailability.

## MATERIALS AND METHODS

### Animals

48 adult male Sprague–Dawley rats (average weight approximately 220±10 g) were purchased from Beijing HFK Bioscience Co., Ltd. All rats were housed with a standard 12-hour light-dark cycle at a temperature of 22±2° C and humidity of 40%-60%. Rats were given free access to standard food and water. Throughout the research, we spare no effort to alleviate the suffering of animals and use as few animals as possible.

### Induction of diabetes and treatment groups

In this 12-week study, a total of 48 rats were randomly divided into 4 groups of 12 each by simple randomization method using Excel software: negative control (NC), NC treated with aspirin (NC+Aspirin), DCP, and DCP treated with aspirin (DCP+Aspirin). Rats in model groups were intraperitoneally injected with STZ (65 mg/kg) to establish a type 1 diabetic rat model [6, 39, 40]. Rats in the control group were intraperitoneally injected with the same dose of citric acid-sodium citrate buffer for the control treatment. After 3 days, blood samples were collected from the tail vein to measure the random blood glucose. A blood glucose level above 16.7 mM confirms the presence of diabetes. Aspirin was dissolved in sterile water and prepared into a concentration of 20 mg/mL. 2 weeks after modeling, the rats in treatment groups received daily aspirin (100 mg/kg/d) by gavage, served with the equal volume control irrigation sterile water (5mL/kg/d) [26, 29, 41]. Subsequently, 12 rats in each group were divided into 3 subgroups according to treatment duration: 3 rats in the 4W group, 3 rats in the 7W group and the remaining 6 rats in the 10W group. After treatment, animals were euthanized in carbon dioxide tanks and bladder specimens were collected.

### Morphological evaluation

The body weights were recorded daily until the ending point. After 10 weeks of administration, random blood glucose was measured before execution. And then we did the gross morphology observation. After macroscopic examination, bladders were rapidly dissected to drain the urine and then weigh the bladder in an empty state. Subsequently, the bladders were opened at the dorsal side of the bladder neck along with the midline towards the dome and then divided longitudinally into muscle strips. DSM strips (5-7 mm long and 2-3 mm wide) from the urinary bladder dome were collected. Half of them removed the bladder mucosa under a stereomicroscope and preserved in liquid nitrogen. The other half of the urinary bladder was placed in a 4% paraformaldehyde solution or 2.5% glutaraldehyde solution for histological.

### H&E, Masson and immunohistochemical staining

The sections were stained with H&E and Masson’s trichrome, then examined by light microscope. For HE staining, sections were randomly selected and scored based on inflammation and infiltration level of the tissue, the severity of edema and hemorrhage. As shown in Supplementary Table 1 [42], the scale ranged from 0 to 5 points indicating light to severe according to the inflammation. The thickness of the bladder wall and the ratio of smooth muscle to collagen within the bladder DSM layer wall were measured by using Masson trichrome-stained images.

For immunohistochemistry (IHC) staining, the tissue sections were incubated with TNF-α (1:100), IL-1β (1:100), NF-κB (1:250), IL-6 (1:100) and β-actin (1:500) primary antibody at 4° C overnight. We observed the five visual fields of every section under a low-power microscope, taking the average value as the observation result of each section. The average optical density was measured by using the IHC Profiler plugin to analyze the images of the sections in Image J (NIH, Bethesda, MD, USA). To minimize the variability between images, the density was normalized to that of an unstained area, and the exposure time and microscope settings were fixed throughout the acquisition.

### Transmission electron microscopy

The bladder specimens were quickly sliced into 1 mm^3^ and immersed in 2.5% glutaraldehyde solution for 48 h. Subsequently, postfixation was performed with 1% osmium tetroxide for 2 h, and dehydration followed by impregnation with epoxy resin was performed. The ultrathin sections were made at 70~80nm and stained with 2% uranyl acetate and Reynolds lead citrate solution. The sections were observed under a transmission electron microscope (H-7700; Hitachi High-Technologies, Tokyo, Japan).

### Quantitative reverse transcription-polymerase chain reaction (qRT-PCR)

The total RNA extraction from DSM tissues was performed using RNAiso Plus Kit (Takara-Bio, Shiga, Japan) according to the manufacturer’s instructions. Reverse transcription of total RNA was performed using PrimeScript RT Master Mix (Takara-Bio, Shiga, Japan). Quantitative reverse-transcription PCR was performed using TB Green Premix Ex TaqTM (Takara-Bio, Shiga, Japan) on an ABI PRISM7500 sequence detection system. The PCR conditions were 95° C for 30 s followed by 40 cycles of 95° C for 5 s and 60° C for 30 s. All of the reactions were run three times, and mRNA expression was normalized relative to GAPDH. Supplementary Figure 2 showed the stability of GAPDH expression. The primer sequences are shown in Table 1.

**Table 1:** Primers sequences.

**Gene**	**Forward**	**Reverse**
NF-κB	5′-GCAAACCTGGGAATACTTCATGTGACTAAG-3′	5′-ATAGGCAAGGTCAGAATGCACCAGAAGTCC-3′
IL-1β	5′-TGACCCATGTGAGCTGAAAG-3′	5′-GGGATTTTGTCGTTGCTTGT-3′
TNF-α	5′-TACTGAACTTCGGGGTGATTGGTCC-3′	5′-CAGCCTTGTCCCTTGAAGAGAACC-3′
IL-6	5′-CTTCCATCCAGTTGCCTTCTTG-3′	5′-AATTAAGCCTCCGACTTGTGAAG-3′
GAPDH	5′-GTTACCAGGGCTGCCTTCTC-3′	5′-ACCAGCTTCCCATTCTCAGC-3′

### Western blot assay

DSM tissues were extracted with RIPA lysis buffer (Solarbio, Beijing, China). Protein concentrations were determined using BCA Protein Quantification Kit (Yeasen Biotech Co., Ltd). The protein sample (20 μg) was loaded onto 12% SDS-PAGE gel for electrophoretic separation and then transferred to a nitrocellulose membrane. The membrane was blocked with Tris-buffered saline with Tween 20 (TBST) containing 5% bovine serum albumin (BSA) for 1.5 h. After shaking slowly at room temperature for 1.5 h, the membrane was incubated with primary rabbit antibody against TNF-α (1:500; Abcam Cat# ab205587, RRID: AB_2889389), IL-1β(1:1000; Novus Cat# NB600-633, RRID: AB_10001060), NF-κB (1:1000; Abcam Cat# ab32360, RRID: AB_776748), and β-actin (1:5000; Abcam Cat# ab8227, RRID: AB_2305186) and with primary mouse antibody against IL-6 (1:1000; Abcam Cat# ab9324, RRID: AB_307175) on a 4° C shaking table overnight. Take out the membrane the next day and clean the membrane 3 times with TBST for 5 minutes each time. Then the membrane was slowly incubated with the secondary antibody (1:5000; Abcam) at room temperature for 1.5h. Subsequently, the PierceTM ECL Western Blotting Substrate Kit (Thermo Fisher Scientific, USA) was used to detect the protein expression. Supplementary Figure 3 showed the original protein bands.

### Statistical analysis

Data were further analyzed with GraphPad Prism 9.0.0 software (San Diego, CA, USA) and were expressed as the mean ± SD. The histological score was assessed using a Kruskal-Wallis test followed by Dunn’s multiple comparisons test. The other data were analyzed with ANOVA followed by the Tukey’ s multiple comparisons test. All images were processed and analyzed using dedicated software (Image Pro 6.0). All microscopic sections were randomly selected and reviewed by two pathologists, who were blinded to the study design. Percentage change of the DCP group from the NC group or percentage change of the DCP+Aspirin group from the DCP group. n=the number of strips or PCR samples and N=the number of rats. P < 0.05 was considered statistically significant.

### Data availability statement

The data generated during and/or analyzed during the current study are available from the corresponding author on reasonable request.

## Supplementary Material

Supplementary Figures

Supplementary Table 1
