# Supplementary material for: Long-term aspirin administration suppresses inflammation in diabetic cystopathy
Source: Aging (Albany NY). 2023 Sep 12;15(17):9128–43. doi: 10.18632/aging.205021 (PMC10522387; doi:10.18632/aging.205021)
Supplement: Supplementary Figures [file aging-15-205021-s001.pdf]

SUPPLEMENTARY FIGURES

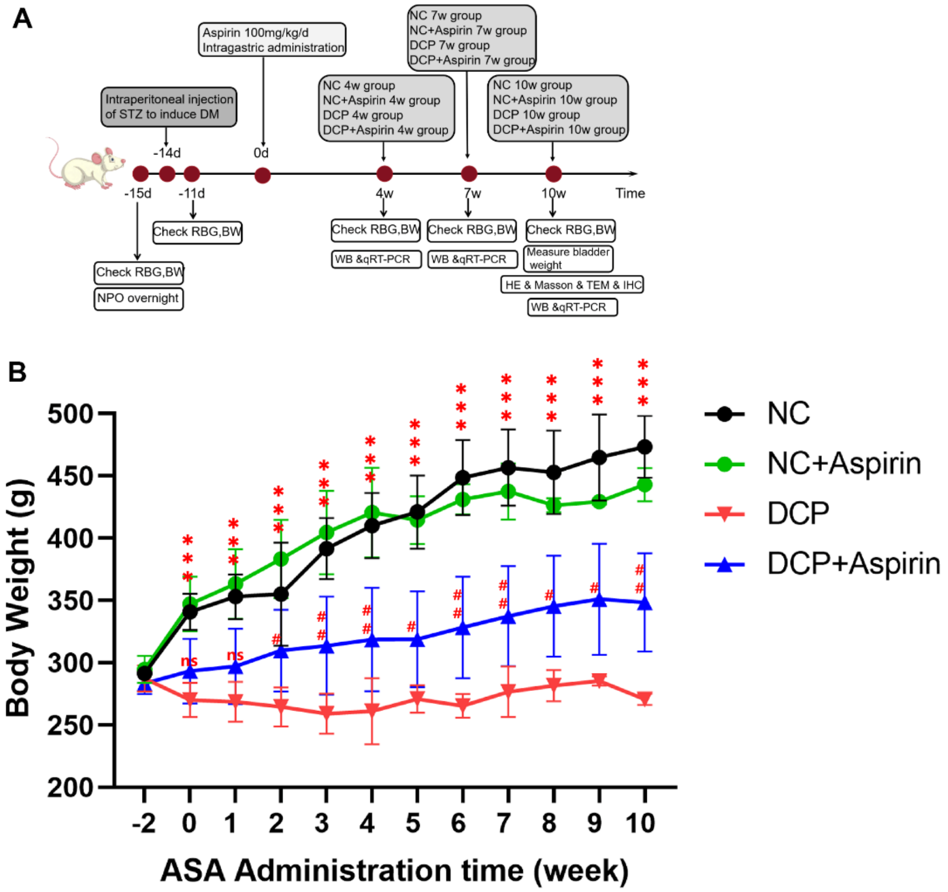

Supplementary Figure 1. (**A**) The detailed flowsheet of the experimental design (**B**) The line chart of body weight displays trend over time. Data were presented as mean ± SD. (ns, no significant; */#, P < 0.05; **/##, P < 0.01; ***/###, P < 0.001; *, NC vs DCP; #, DCP vs DCP+Aspirin).

A

Groups（mean±SD）	4W	7W	10W
NC	16.39±0.49	16.17±0.39	16.72±0.46
NC+Aspirin	16.48±0.32	16.48±0.32	16.47±0.68
DCP	16.24±0.28	16.18±0.55	16.47±0.89
DCP+Aspirin	16.22±0.40	15.99±0.37	16.52±0.73

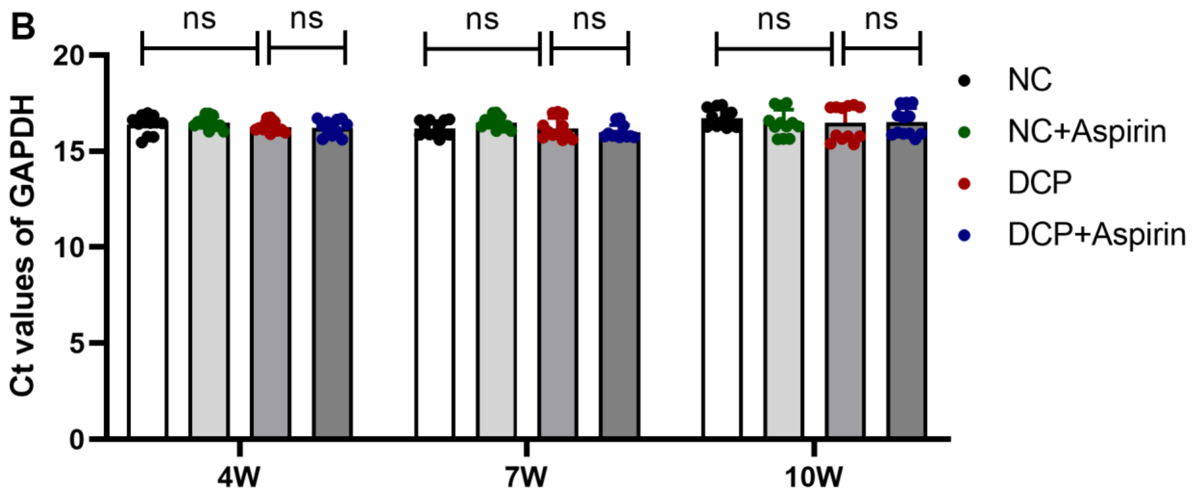

Supplementary Figure 2. Stability of reference gene GAPDH in mRNA analysis of rat bladder. (A) Ct values of GAPDH were presented as mean ± SD. (B) Statistical analysis of GAPDH expression. n=12. N=6. (ns, no significant; NC vs DCP; DCP vs DCP+Aspirin).

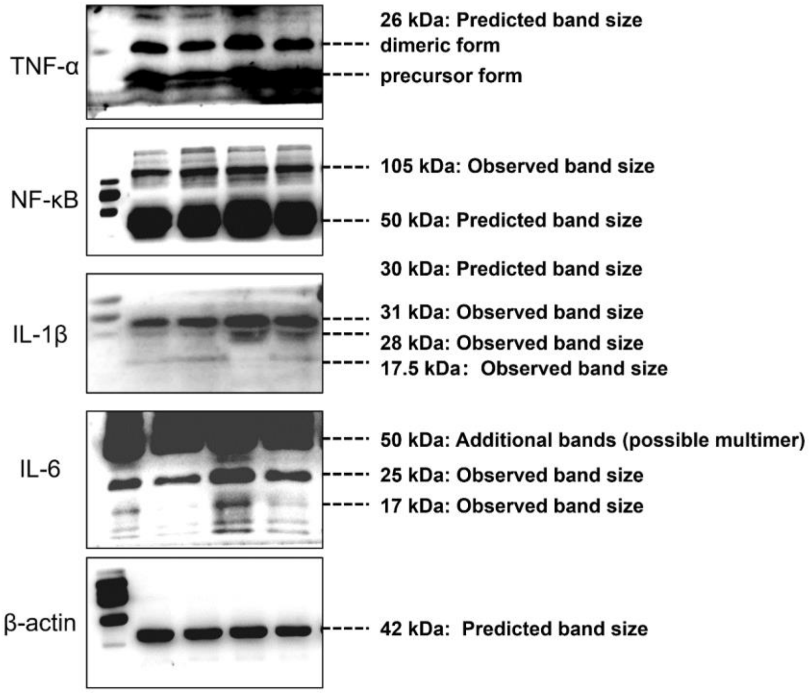

Supplementary Figure 3. The original Western blot bands show a detailed characterization demonstrating the specificity of the antibody and the range of reactivity of the reagent in the assay.
