# Supplementary material for: Long-term aspirin administration suppresses inflammation in diabetic cystopathy
Source: Aging (Albany NY). 2023 Sep 12;15(17):9128–43. doi: 10.18632/aging.205021 (PMC10522387; doi:10.18632/aging.205021)
Supplement: Supplementary Table 1 [file aging-15-205021-s002.pdf]

SUPPLEMENTARY TABLE

Supplementary Table 1. Histology score based on inflammation (From the reference [42]).

Histology score based on inflammation	
**Score**	**Histological characteristics**
0	Morphologically unremarkable with no or very minimal inflammation or epithelial changes.
1	Minimal inflammatory infiltrate composed of occasional neutrophils or lymphocytes within the lamina propria in the absence of inflammation in the muscularis propria, or significant edema, hemorrhage or urothelial changes.
2	Minimal to mild inflammatory infiltrate within the lamina propria with scattered neutrophils or lymphocytes, accompanied by mild edema or hemorrhage, but in the absence of inflammation in the muscularis propria or significant urothelial changes.
3	Mild or mild to moderate inflammatory infiltrate in the lamina propria and focal extension of the inflammation into the muscularis propria.
4	Moderate inflammation with scattered to frequent neutrophils and lymphocytes in both the lamina propria and muscularis propria.
5	Severe inflammation in the lamina propria and muscularis propria in association with other significant findings, such as urothelial ulceration, severe edema, hemorrhage and fibrin deposition.
